# Supplementary material for: Komagataella phaffii Encodes Two Functional Pho4 Transcription Factors
Source: Int J Mol Sci. 2026 Jul 23;27(15):6561. doi: 10.3390/ijms27156561 (PMC13466545; doi:10.3390/ijms27156561)
Supplement: Supplementary file 1 [file ijms-27-06561-s001.zip › Supplementary Table S4.pdf]

Supplementary Table S4. *K. phaffii* strains used in this work.

Strain	Recipient strain	mutated gene(s)	sgRNA	Clone	Genotype	Mutation and effect on the protein	Reference
**PJA-004**	X-33	*PHO4(A)* (PAS_chr1-1_0265)	Indel_1	3.1	*pho4(A)Δ110*	Deletion of one C at position 110, change of frame after His36 and stop appears after codon 57	this work
**PJA-005**			Indel_6	4.1	*pho4(A)-111*	Insertion of one A after position 111, change of frame after Pro37 with premature stop after codon 46	this work
**PJA-021**	X-33	*PHO4(B)* (PAS_chr2-1_0177)	Indel_1	P1A	*pho4(B)Δ314-315*	Deletion of 2 bases (TG) at position 314-315, change of frame after Leu 105 with premature stop after codon 149	this work
**PJA-022**			Indel_2	P2B	*pho4(B)-117*	Insertion of one T after position 116, change of frame after Thr38 and appears stop after codon 43	this work
**PJA-026**	PJA-004	*PHO(A) PHO4(B)*	Indel_1	P5B	*pho4(A)Δ110 pho4(B)-315*	Insertion of one T after position 314, change of frame after Leu105 and stop appears after codon 150	this work
**PJA-035**			Indel_2	P6B-4	*pho4(A)Δ110 pho4(B)Δ115*	Deletion of one A at position 115, creates a stop right after deletion point (after Thr 38)	this work
**PJA-036***			Indel_2	P6B-5	*pho4(A)Δ110 pho4(B)Δ116*	Deletion of one A at position 116, change of frame after Thr38 and appears stop after codon 70	this work
**PJA-037**	X-33	none			*pPHO89:GFP*	None	[1]
**PJA-038**	PJA-004	*PHO4(A)*			*pho4(A)Δ110 pPHO89:GFP*	Deletion one C at position 110	this work
**PJA-039**	PJA-021	*PHO4(B)*			*pho4(B)Δ314-315 pPHO89:GFP*	Deletion of 2 bases (TG) at position 314-315	this work
**PJA-040**	PJA-026	*PHO(A) PHO4(B)*			*pho4(A)Δ110 pho4(B)-315 pPHO89:GFP*	Insertion of one T after position 314	this work

* This strain was used as template for amplification of the mutated *KpPHO4(B)* allele for expression in *S. cerevisiae*

1. Albacar, M. *et al.* Transcriptomic profiling of the yeast *Komagataella phaffii* in response to environmental alkalinization. *Microb. Cell Fact.* **22**, 63 (2023).
