# Supplementary material for: Komagataella phaffii Encodes Two Functional Pho4 Transcription Factors
Source: Int J Mol Sci. 2026 Jul 23;27(15):6561. doi: 10.3390/ijms27156561 (PMC13466545; doi:10.3390/ijms27156561)
Supplement: Supplementary file 1 [file ijms-27-06561-s001.zip › Supplementary Figs S1-S6.pdf]

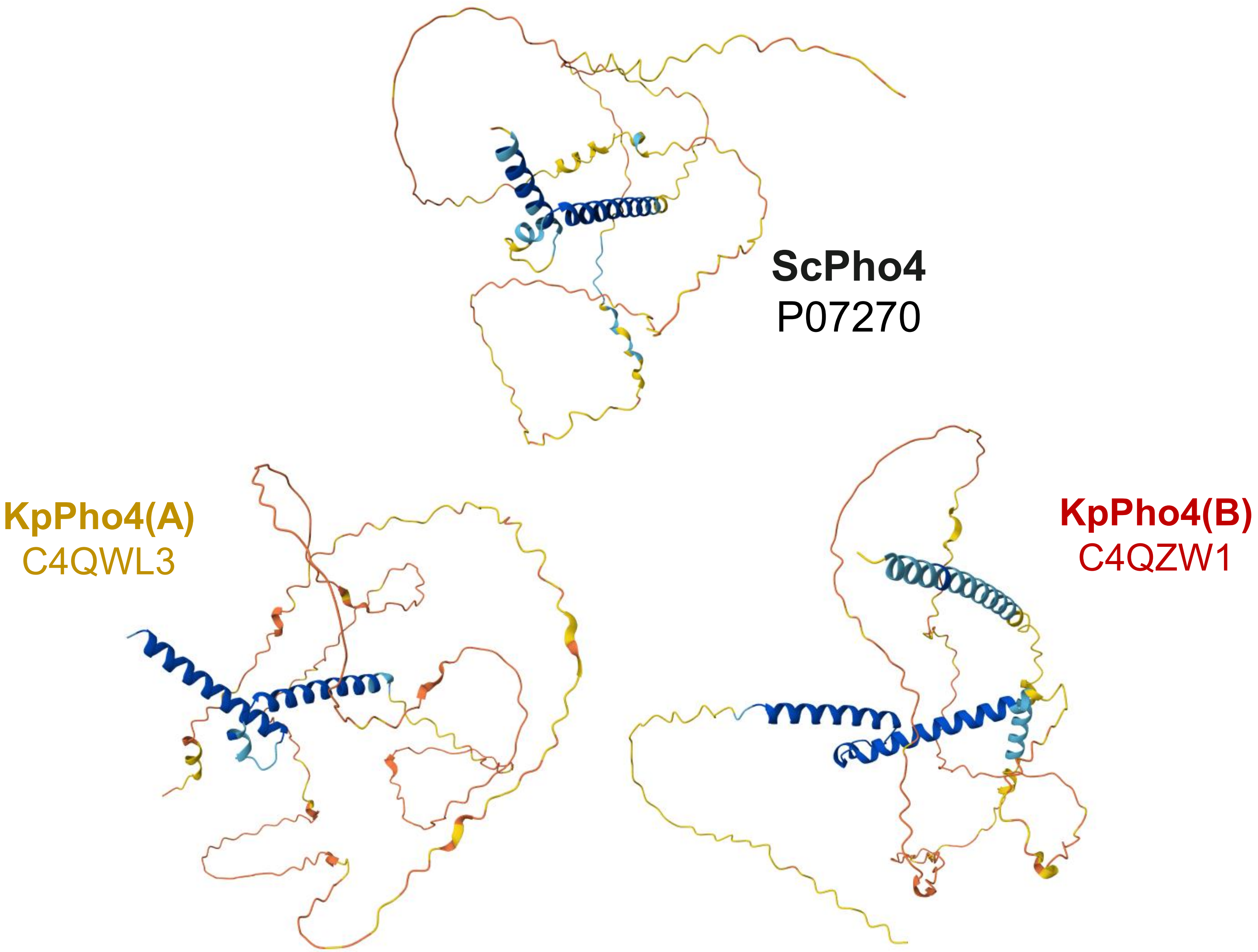

Supplementary Fig S1. AlphaFold predicted structures for *S. cerevisiae* Pho4 (ScPho4) and the putative KpPho4(A) and KpPho4(B) homologs in *K. phaffii*. Created with the AlphaFold Monomer v2.0 pipeline. Last updated in AlphaFold DB version 2022-11-01. Uniprot entry codes are provided for each protein. Note the N-terminal α–helix predicted for KpPho4(B) and not present in ScPho4 or KpPho4(A).

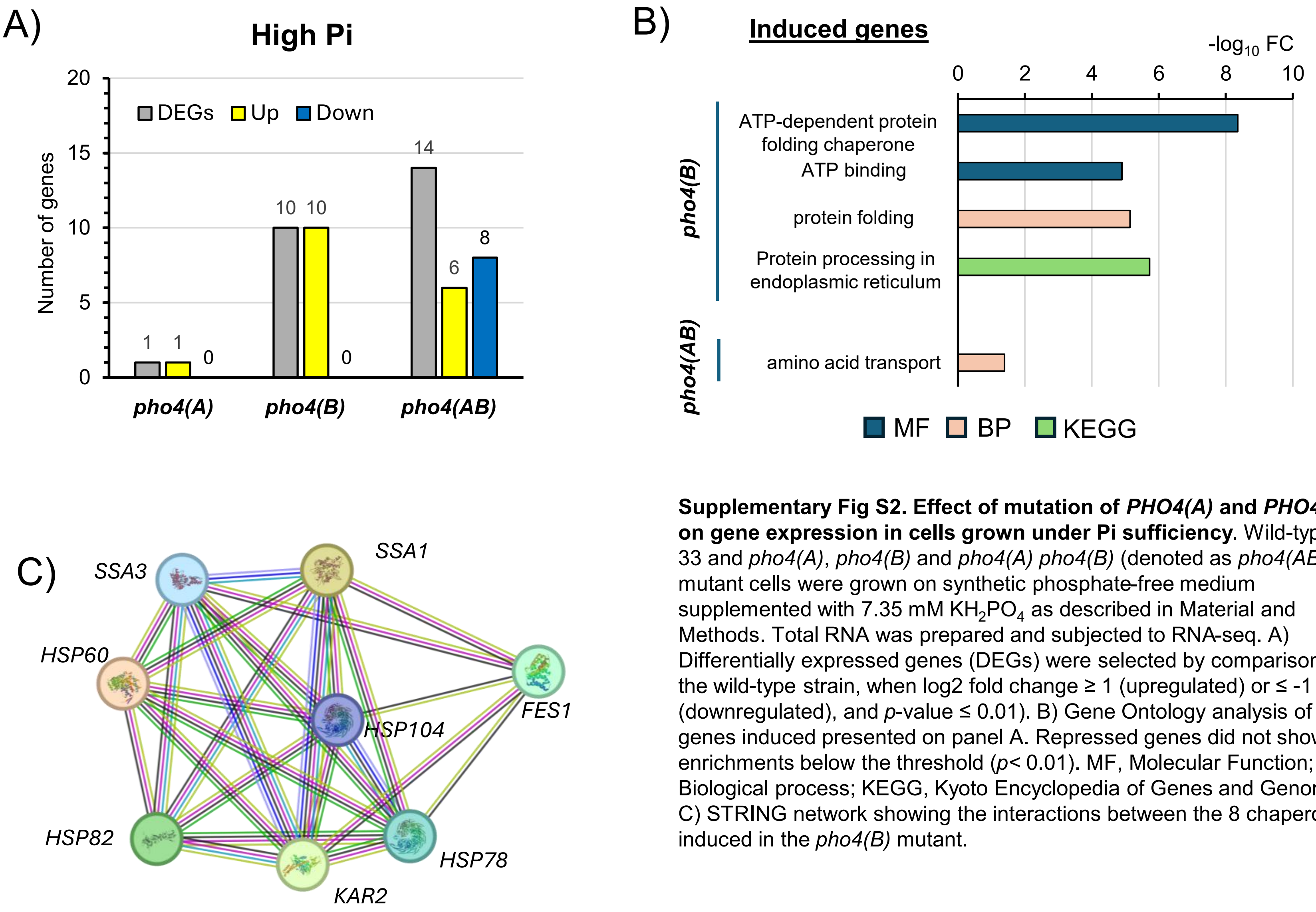

Supplementary Fig S2. Effect of mutation of *PHO4(A)* and *PHO4(B)* on gene expression in cells grown under Pi sufficiency. Wild-type X-33 and *pho4(A)*, *pho4(B)* and *pho4(A) pho4(B)* (denoted as *pho4(AB)*) mutant cells were grown on synthetic phosphate-free medium supplemented with 7.35 mM KH_2PO_4 as described in Material and Methods. Total RNA was prepared and subjected to RNA-seq. A) Differentially expressed genes (DEGs) were selected by comparison with the wild-type strain, when log2 fold change ≥ 1 (upregulated) or ≤ -1 (downregulated), and *p*-value ≤ 0.01). B) Gene Ontology analysis of the genes induced presented on panel A. Repressed genes did not show enrichments below the threshold (p< 0.01). MF, Molecular Function; BP, Biological process; KEGG, Kyoto Encyclopedia of Genes and Genomes. C) STRING network showing the interactions between the 8 chaperones induced in the *pho4(B)* mutant.

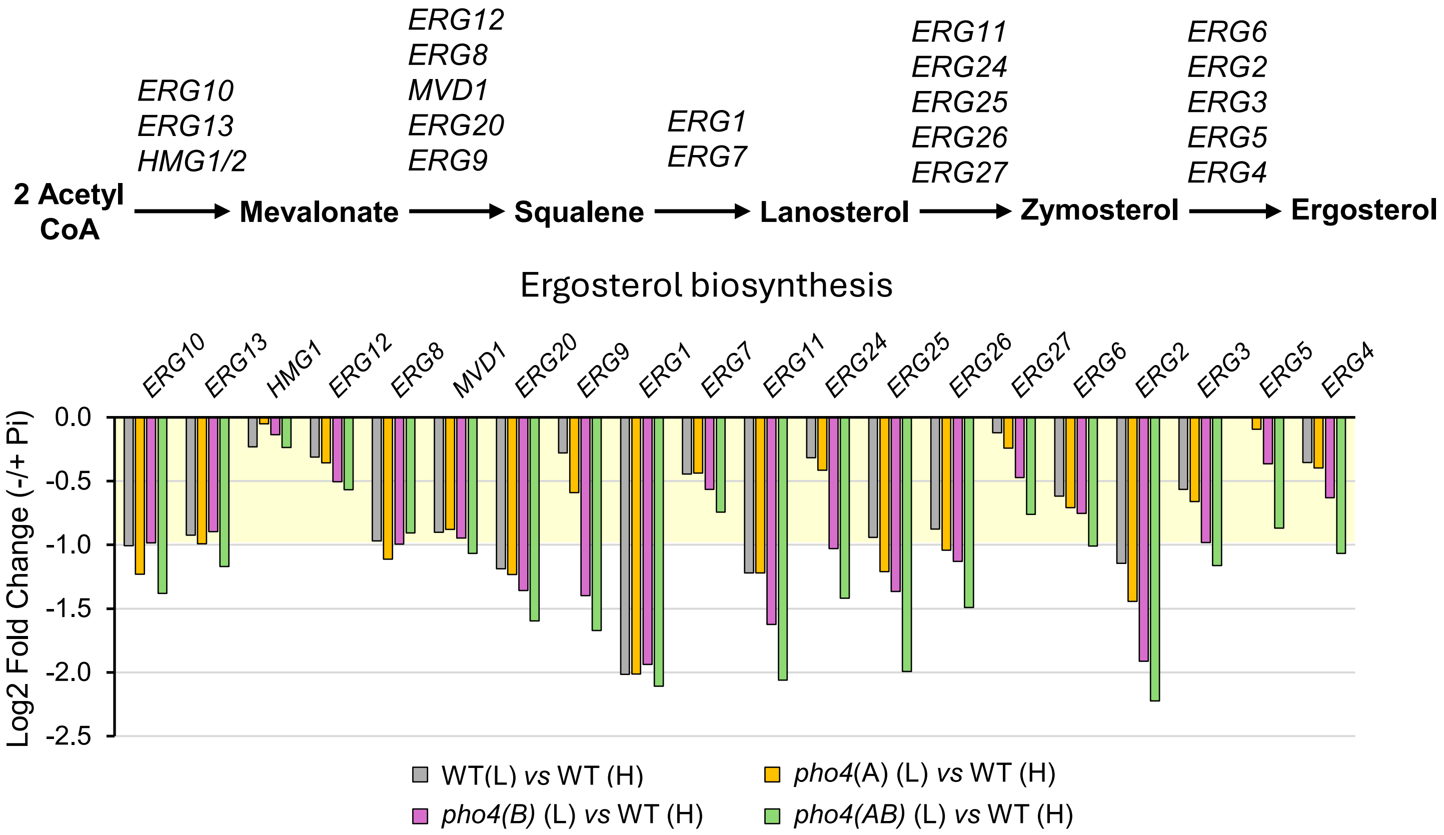

Supplementary Fig S3. Changes in the expression of the genes involved in ergosterol biosynthesis in response to phosphate starvation in wild type, *pho4(A)*, *pho4(B)* and *pho4(A) pho4(B)* (denoted as *pho4(AB)*) strains. Values are log2 fold changes for each strain grown in the absence of Pi (L) *vs* the wild-type strain grown in the presence of Pi (H). The scheme in the upper section reproduces the sequence of genes involved in the synthesis of ergosterol from Acetyl CoA and the yellow strip delimits the log2 FC <-1 area.

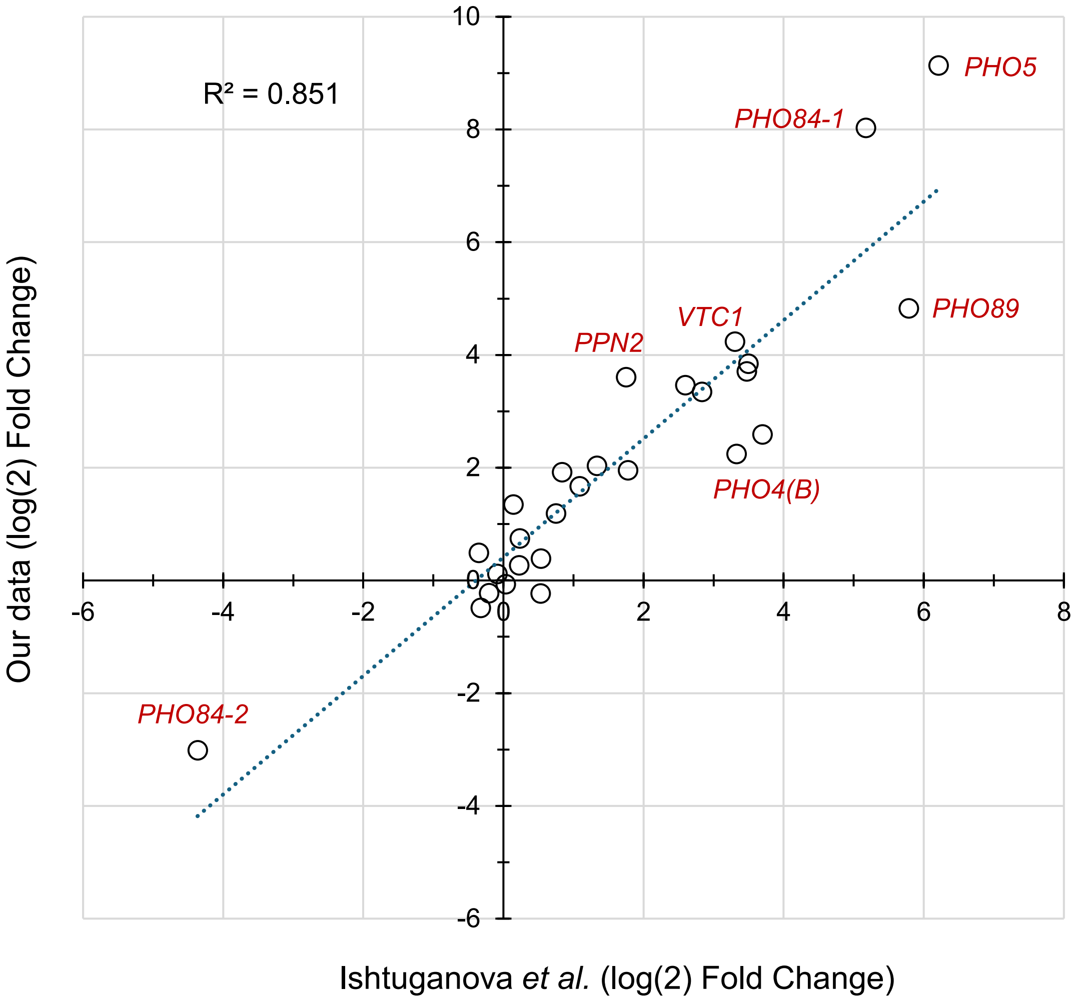

Supplementary Fig S4. Comparison of the response of 28 members of the PHO regulon between our data and that reported by Ishtuganova and coworkers (DOI: 10.3390/microorganisms13010039). Linear regression was calculated and specific genes are labelled.

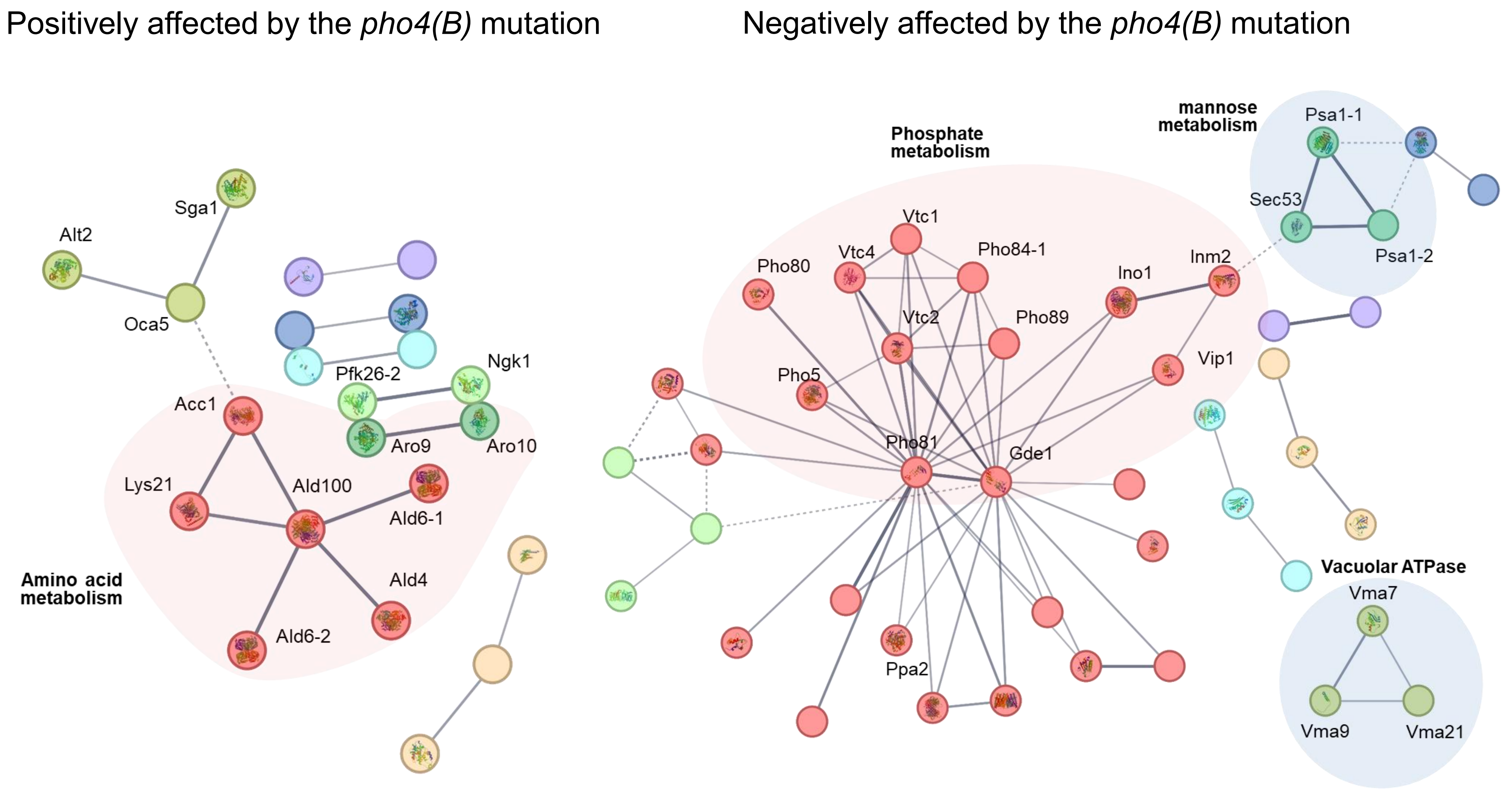

Supplementary Fig S5. STRING analysis of proteins encoded by differentially expressed genes in the *pho4(B)* mutant at the high Pi to low Pi transition. Functionally unconnected proteins were removed for simplicity. Colored background was drawn manually to visualize functional grouping. K-means clustering (8 clusters) was used.

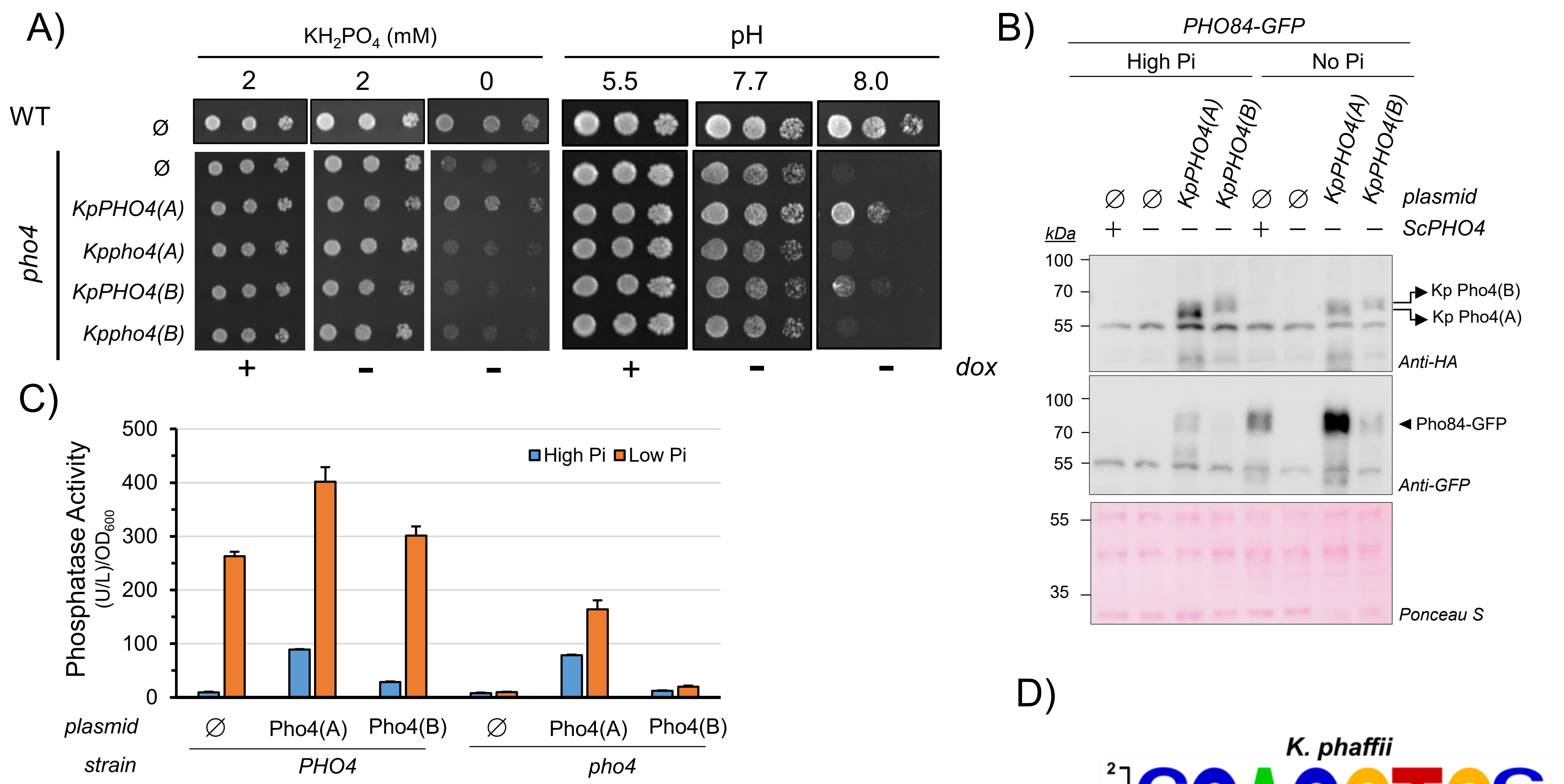

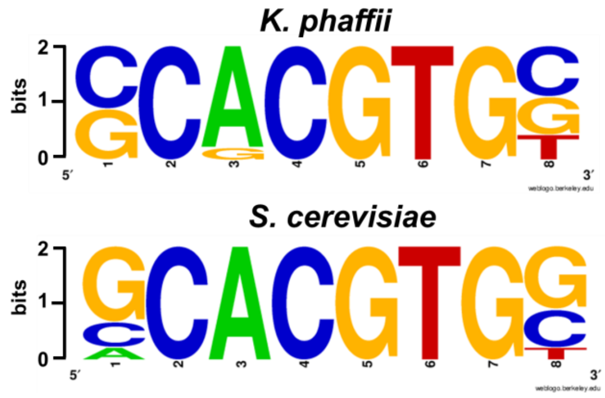

Supplementary Fig S6. Expression of *K. phaffii* Pho4 transcription factors in the *S. cerevisiae* BY4741 genetic background. A) Wild-type strain BY4741 (WT) and its isogenic *pho4* derivative were transformed with the empty vector pCM189 (∅) or the same vector carrying 3xHA-tagged version of *KpPHO4(A)*, *KpPHO4(B)* or their mutated versions (*Kppho4(A), Kppho4(B)*). Transformants were spotted on synthetic medium (Pi free) plates carrying the indicated amounts of Pi or in YPD adjusted at the indicated pH, and growth recorded after 3 days at 28 °C. B) Wild-type BY4741 (+) and its *pho4* derivative (-) were co-transformed with the indicated pCM189-based plasmids plus the YCp111-PHO84-GFP vector. After growth for 20 h in the absence of doxycycline, cells were washed, resuspended in medium with or without Pi and growth resumed. Samples were taken after 2 h and extracts (10 µg of protein) were subjected to10% PAGE, transferred to membranes and probed with anti-GFP and anti-HA antibodies. C) Strain BY4741 (*PHO4*) and its *pho4* derivative were transformed with the indicated plasmids and processed for secreted phosphatase activity determination as in Figure 7. Data are mean ± SEM from four biological replicates determined in duplicate. D) Graphical representation of the consensus pattern found in the upstream regions of the genes *PHO81*, *PHO5*, *PHO4*(B), *PHO89*, *VTC2*, *VTC4*, *VTC1*, *PHO84-1*, and *PHO86* in *K. phaffii* and *S. cerevisiae*.
