# Supplementary material for: Komagataella phaffii Encodes Two Functional Pho4 Transcription Factors
Source: Int J Mol Sci. 2026 Jul 23;27(15):6561. doi: 10.3390/ijms27156561 (PMC13466545; doi:10.3390/ijms27156561)
Supplement: Supplementary file 1 [file ijms-27-06561-s001.zip › Supplementary Methods S1.pdf]

Komagataella phaffii encodes two functional Pho4 transcription factors

Marcel Albacar, Asier González, Rui Wang, Antonio Casamayor, Joaquín Ariño

Supplementary Methods

Plasmids construction for expression in *S. cerevisiae*

For expression of Pho4(A) and Pho4(B) these ORFs were initially cloned into the high-copy plasmid pWS93 [1], which carries the *URA3* marker and allows expression of N-terminally 3x-HA tagged proteins from the strong *ADH1* promoter. To this end, the ORFs were amplified from genomic DNA with the oligonucleotide pairs pho4(A)-expSc-5 / Pho4(A)-expSc-3 and Pho4(B)-expSc-5 / Pho4(B)-expSc-3, respectively, digested with EcoRI and BamHI, and ligated into these sites present in the vector. Plasmid pCM189 was used for regulated expression of the ORFs. This centromeric *tet-off* vector carries the *URA3* marker and allows expression when cells are grown in the absence of doxycycline [2]. The expression vectors were made as follows. The *PHO4(A)* and *PHO4(B)* ORFs, including the 3x-HA NH_2-terminal tag, were amplified from pWS93-PHO4(A) and pWS93-PHO4(B), respectively, with the pairs of primers 3xHA-tetO-5 / Pho4(A)-tetO-3 and 3xHA-tetO-5 / Pho4(B)-tetO-3. The 3x-HA-PHO4(A) amplification fragment was digested with BamHI and PstI, whereas the 3x-HA-PHO4(B) sequence was treated with BamHI and NotI, and both fragments were cloned into the pCM189 vector linearized with the appropriate enzymes. The same procedures were applied to amplify and clone the mutated versions of *PHO4(A)* and *PHO4(B)* from strains PJA-004 (*pho4(A)*Δ110) and PJA-036 (*pho4(B)*Δ116), respectively. All amplifications were carried out with Q5 DNA polymerase (New England Biolabs) and the final products were sequenced to assess unwanted mutations due to PCR amplification. Oligonucleotides employed can be found at Supplemental_Table_S1A.

The pPHO84-GFP reporter, which expresses green fluorescent protein (GFP) from the endogenous *PHO84* promoter was constructed by removing a 3.06 kbp BamHI/XhoI fragment containing the PHO84 promoter and ORF plus a C-terminal GFP fusion from plasmid EB0666 described in (Lau et al., 2000). This fragment was cloned into the YCplac111 (*CEN*, *LEU2*) vector [3] previously digested with BamHI and SalI to yield plasmid YCp111-Pho84-GFP.

Immunoblotting

Whole cell extracts were prepared essentially as described in [4] with some modifications. Cells were lysed in 150 µl of lysis buffer (50 mM Tris·HCl, pH 7.5, 0.5%

Triton X-100, 10% (vol/vol) glycerol, 1 mM dithiothreitol (DTT), 0.2 mM phenylmethylsulfonyl fluoride (PMSF), phosphatase inhibitor cocktail set V (Calbiochem) and complete protease inhibitor mixture (Roche)) and the same volume of 0.5 mm diameter glass beads (BioSpec). Tubes were vortexed 10 times for 20 s (with intervals of 20 s on ice after each vortexing round) and centrifuged at 2000 xg for 1 min at 4 ºC. Supernatants were collected in fresh 1.5 ml tubes pre-chilled on ice and further clarified by centrifugation at 8000xg for 5 min at 4 ºC. Protein amount was quantified with Coomassie Plus protein assay reagent (Thermo Scientific). Proteins (10 µg) were separated by electrophoresis on 10 % SDS-polyacrylamide gels. The protein ladder PageRuler Plus (Thermo Scientific) was used as a molecular weight marker. Proteins were transferred onto polyvinylidene difluoride (PVDF) membranes (Immobilon-P, Millipore) and analyzed by immunoblot using Anti-HA.11 epitope tag antibody (Cat. No. 901513, BioLegend) or anti-GFP antibody (Cat. No. 11814460001, Roche) at 1:1000 dilution followed by ECL anti-mouse IgG–horseradish peroxidase (HRP) secondary antibodies (GE Healthcare) at 1:10000 dilution. Proteins were visualized using the Westar ηc ultra 2.0 chemiluminiscence substrate (Cyanagen) on an ODYSSEY® XF apparatus (LICORbio). For membrane stripping, membranes were incubated in stripping solution (15 g/l glycine, 1 g/l SDS, 0.1 % Tween-20, pH 2.2) for 10 min, washed 2 times with TBST (TBS plus 0.1% Tween-20), and reprobed as required. Ponceau S staining was used as loading control.

Other techniques

Evolutionary analysis of fungal Pho4 proteins was made by the Maximum Likelihood method with the MEGA12 software and default settings [5]. Identification of clusters of acidic and hydrophobic residues to predict transcriptional activation domains [6] was performed with an in-house algorithm. Search for putative Pho4 binding sites was made with the RSAT platform by mining the upstream -800/-1 sequences (relative to the initiating ATG and allowing overlapping with adjacent CDS) from the *K. phaffii* GS115 genome (GCF_000027005.1_ASM2700v1) from selected genes. The Quick Matrix-Scan algorithm [7] with a *p*-value of 10^{-4} was set as selection threshold. Logos for TF consensus binding sequences were made with the WebLogo platform (v2.8.2) [8].

References

1. Song, W. & Carlson, M. Srb/mediator proteins interact functionally and physically with transcriptional repressor Sfl1. *EMBO J.* **17**, 5757–5765 (1998).

2. Gari, E., Piedrafita, L., Aldea, M. & Herrero, E. A set of vectors with a tetracycline-regulatable promoter system for modulated gene expression in Saccharomyces cerevisiae. *Yeast* **13**, 837–848 (1997).

3. Gietz, R. D. & Sugino, A. New yeast-Escherichia coli shuttle vectors constructed with in vitro mutagenized yeast genes lacking six-base pair restriction sites. *Gene* **74**, 527–534 (1988).

4. Ruiz, A., Xu, X. & Carlson, M. Roles of two protein phosphatases, Reg1-Glc7 and Sit4, and glycogen synthesis in regulation of SNF1 protein kinase. *Proc. Natl. Acad. Sci. U.S.A* **108**, 6349–6354 (2011).

5. Kumar, S. *et al.* MEGA12: Molecular Evolutionary Genetic Analysis Version 12 for Adaptive and Green Computing. *Mol. Biol. Evol.* **41**, msae263 (2024).

6. Kotha, S. R. & Staller, M. V. Clusters of acidic and hydrophobic residues can predict acidic transcriptional activation domains from protein sequence. *Genetics* **225**, iyad131 (2023).

7. Turatsinze, J. V., Thomas-Chollier, M., Defrance, M. & van Helden, J. Using RSAT to scan genome sequences for transcription factor binding sites and cis-regulatory modules. *Nat. Protoc.* **3**, 1578–1588 (2008).

8. Crooks, G. E., Hon, G., Chandonia, J. M. & Brenner, S. E. WebLogo: a sequence logo generator. *Genome Res.* **14**, 1188–1190 (2004).
