# Supplementary material for: Komagataella phaffii Encodes Two Functional Pho4 Transcription Factors
Source: Int J Mol Sci. 2026 Jul 23;27(15):6561. doi: 10.3390/ijms27156561 (PMC13466545; doi:10.3390/ijms27156561)
Supplement: Supplementary file 1 [file ijms-27-06561-s001.zip › Supplementary Table S1.pdf]

Supplementary Table S1

Title:NCBI Protein Reference Sequences

Molecule Type:Protein

Update date:2025/07/22

Number of sequences:406273733

Search Parameters	
Program	blastp
Word size	5
Expect value	0.01
Hitlist size	250
Gapcosts	11,1
Matrix	BLOSUM62
Filter string	F
Genetic Code	1
Window Size	40
Threshold	0
Composition-based stats	2

Komagataella phaffii GS115 hits are in yellow background

Description	Scientific Name	Taxid	Max Score	Total Scoı	Query Cov	E value	Per. ident	Acc. Len	NCBI Accession
phosphate-sensing transcription factor PHO4	Saccharomyces cerevisiae S288C	559292	640	640	100%	0	100	312	NP_116692.1
phosphate-sensing transcription factor PHO4 SKDI_06G1040	Saccharomyces kudriavzevii IFO 1802	226230	510	510	100%	3.00E-180	84.47	316	XP_056087509.1
phosphate-sensing transcription factor PHO4	Saccharomyces paradoxus	27291	507	507	100%	3.00E-179	89.91	314	XP_033766099.1
phosphate-sensing transcription factor PHO4 SMKI_07G2970	Saccharomyces mikatae IFO 1815	226126	492	492	100%	4.00E-173	81.79	321	XP_056082429.1
phosphate-sensing transcription factor PHO4 partial	Saccharomyces eubayanus	1080349	280	280	73%	1.00E-90	78.26	236	XP_018222570.1
phosphate-sensing transcription factor PHO4	Eremothecium gossypii ATCC 10895	284811	83.6	83.6	40%	6.00E-14	44.85	396	NP_983973.3
phosphate-sensing transcription factor PHO4	Lachancea thermotolerans CBS 6340	559295	84	84	39%	7.00E-14	42.21	466	XP_002553686.1
phosphate-sensing transcription factor PHO4 LALA0_S01e15016g	Lachancea lanzarotensis	1245769	80.1	80.1	38%	1.00E-12	35.59	547	XP_022626857.1
uncharacterized protein KUCA_T00001297001	Kuraishia capsulata CBS 1993	1382522	75.1	75.1	36%	8.00E-11	42.74	573	XP_022457340.1
phosphate-sensing transcription factor PHO4	Nakaseomyces bracarensis	273131	74.7	74.7	38%	9.00E-11	32.5	488	XP_070911366.1
phosphate-sensing transcription factor PHO4 PWA37_005333	Arxiozyma heterogenica	278026	73.9	73.9	21%	2.00E-10	59.38	503	XP_064716733.1
Hypothetical protein PAS_chr1-1_0265	Komagataella phaffii GS115	644223	72.4	72.4	38%	4.00E-10	39.2	380	XP_002489917.1
phosphate-sensing transcription factor PHO4 SCDLUD_002462	Saccharomycodes ludwigii	36035	72	72	36%	9.00E-10	44.35	797	XP_045934930.1
phosphate-sensing transcription factor PHO4 KNAG_0L01210	Huiozyma naganishii CBS 8797	1071383	70.5	70.5	30%	2.00E-09	43.62	476	XP_022466985.1
phosphate-sensing transcription factor PHO4 KABA2_03S11242	Maudiozyma barnettii	61262	70.5	70.5	20%	3.00E-09	57.14	590	XP_041405890.1
phosphate-sensing transcription factor PHO4	Eremothecium sinecaudum	45286	69.7	69.7	38%	4.00E-09	38.78	493	XP_017987657.1
phosphate-sensing transcription factor PHO4 TDEL_0H02560	Torulaspora delbrueckii	4950	68.9	68.9	44%	7.00E-09	32.37	485	XP_003683326.1
phosphate-sensing transcription factor PHO4	Candidozyma auris	498019	68.9	68.9	35%	7.00E-09	41.23	469	XP_028891913.2
phosphate-sensing transcription factor PHO4	Nakaseomyces glabratus	5478	68.6	68.6	23%	1.00E-08	49.33	533	XP_445634.1
PHO4	[Candida] subhashii	561895	67.4	67.4	24%	3.00E-08	48.1	560	XP_049262169.1
hypothetical protein PICMEDRAFT_18182	Pichia membranifaciens NRRL Y-2026	763406	66.2	66.2	20%	7.00E-08	53.12	734	XP_019015914.1
myc-family transcription factor	Scheffersomyces xylosifermentans	1304137	65.9	65.9	22%	8.00E-08	48	597	XP_064777409.1
uncharacterized protein ASCRUDRAFT_5375	Ascoidea rubescens DSM 1968	1344418	65.5	65.5	29%	1.00E-07	40.43	813	XP_020049678.1
phosphate-sensing transcription factor PHO4	Zygosaccharomyces rouxii	4956	65.1	65.1	18%	1.00E-07	60.71	483	XP_002497114.1
phosphate-sensing transcription factor PHO4 Ecym_4385	Eremothecium cymbalariae DBVPG#7215	931890	65.1	65.1	23%	1.00E-07	53.42	580	XP_003646257.1
uncharacterized protein KQ657_004202	Scheffersomyces spartinae	45513	65.1	65.1	29%	1.00E-07	43.33	464	XP_043050633.1
PHO4	Candida metapsilosis	273372	65.1	65.1	20%	2.00E-07	54.84	676	XP_067549196.1
phosphate-sensing transcription factor PHO4 TPHA_0C04480	Tetrapisispora phaffii CBS 4417	1071381	64.7	64.7	18%	2.00E-07	57.89	546	XP_003685033.1
phosphate-sensing transcription factor PHO4 HG536_0C04400	Torulaspora globosa	48254	64.3	64.3	18%	3.00E-07	60.71	504	XP_037138946.1
uncharacterized protein BABINDRAFT_24993 partial	Babjeviella inositovora NRRL Y-12698	984486	58.5	58.5	18%	3.00E-07	50.77	65	XP_018985399.1
Hypothetical protein PAS_chr2-1_0177	Komagataella phaffii GS115	644223	63.5	63.5	20%	3.00E-07	47.54	365	XP_002491065.1
phosphate-sensing transcription factor PHO4 TBLA_0J01560	Henningerozyma blattae CBS 6284	1071380	63.5	63.5	54%	5.00E-07	29.28	714	XP_004182670.1
myc-family transcription factor	Scheffersomyces amazonensis	1078765	63.5	63.5	18%	5.00E-07	55.36	652	XP_064738245.1
uncharacterized protein PGUG_05400	Meyerozyma guilliermondii ATCC 6260	294746	63.2	63.2	19%	5.00E-07	56.9	432	XP_001482380.2
phosphate-sensing transcription factor PHO4 Kpol_1063p15	Vanderwaltozyma polyspora DSM 70294	436907	63.2	63.2	18%	6.00E-07	57.14	587	XP_001643262.1
myc-family transcription factor	Scheffersomyces coipomensis	1788519	63.2	63.2	19%	6.00E-07	53.33	675	XP_064761211.1
phosphate-sensing transcription factor PHO4 HG535_0F03220	Zygotorulaspora mrakii	42260	63.2	63.2	20%	7.00E-07	55.38	618	XP_037145537.1

hypothetical protein CXQ85_002981	Candidozyma haemuli	45357	62.4	62.4	27%	9.00E-07	44.05	477	XP_025344189.1
PHO4	Candida theae	1198502	62.4	62.4	19%	1.00E-06	53.33	676	XP_051609035.1
hypothetical protein CORT_0B00760	Candida orthopsilosis Co 90-125	1136231	62	62	18%	2.00E-06	57.14	620	XP_003867235.1
uncharacterized protein CPAR2_500620	Candida parapsilosis	5480	61.6	61.6	18%	2.00E-06	57.14	638	XP_036667001.1
uncharacterized protein LODBEIA_P40180	Lodderomyces beijingensis	1775926	61.6	61.6	18%	2.00E-06	55.36	738	XP_066830956.1
hypothetical protein C7M61_002624	Candidozyma pseudohaemuli	418784	61.2	61.2	31%	2.00E-06	38.46	440	XP_024713918.1
uncharacterized protein BROUX77_000710	Berkeleyomyces rouxiae	2035830	61.6	61.6	44%	2.00E-06	36	679	XP_073608947.1
uncharacterized protein SPAPADRAFT_58927	Spathaspora passalidarum NRRL Y-27907	619300	61.2	61.2	18%	2.00E-06	55.36	508	XP_007373142.1
PHO4	Candida oxycetoniae	497107	61.2	61.2	18%	3.00E-06	57.14	627	XP_049182379.1
uncharacterized protein CXQ87_003419	Candidozyma duobushaemuli	1231522	60.1	60.1	27%	6.00E-06	44.05	440	XP_025336514.1
PHO4	Candida margitis	1775924	59.3	59.3	19%	1.00E-05	50	615	XP_051670915.1
helix-loop-helix DNA-binding domain-containing protein	Daldinia vernicosa	114800	59.3	59.3	20%	1.00E-05	46.15	685	XP_047860135.1
uncharacterized protein N656DRAFT_713753	Canariomyces notabilis	2074819	59.3	59.3	20%	1.00E-05	45.83	702	XP_064668113.1
Helix-loop-helix DNA-binding domain family protein	Clavispora lusitaniae	36911	57.4	57.4	18%	4.00E-05	53.57	391	XP_002616143.2
Phosphorus acquisition-controlling protein	Colletotrichum siamense	690259	57.8	57.8	20%	4.00E-05	45.95	683	XP_036502496.1
uncharacterized protein COL26b_005148	Colletotrichum chrysophilum	1836956	57.8	57.8	20%	4.00E-05	45.95	643	XP_053038124.1
Phosphorus acquisition-controlling protein	Colletotrichum aenigma	1215731	57.8	57.8	20%	4.00E-05	45.95	683	XP_037186062.1
helix-loop-helix DNA-binding domain-containing protein	Daldinia loculata	103429	57.8	57.8	20%	4.00E-05	44.87	681	XP_049160307.1
Phosphorus acquisition-controlling protein	Colletotrichum truncatum	5467	57.8	57.8	20%	4.00E-05	44.74	687	XP_036585280.2
helix-loop-helix DNA-binding domain-containing protein	Daldinia decipiens	326647	57.8	57.8	20%	4.00E-05	44.87	681	XP_049098615.1
Phosphorus acquisition-controlling protein	Colletotrichum fructicola Nara gc5	1213859	57.4	57.4	20%	5.00E-05	44.59	683	XP_031890683.1
putative phosphorus acquisition-controlling protein	Phaeoacremonium minimum UCRPA7	1286976	57	57	49%	6.00E-05	33.14	578	XP_007914704.1
uncharacterized protein BCR38DRAFT_483489	Pseudomassariella vexata	1141098	57	57	19%	7.00E-05	44.16	682	XP_040716773.1
uncharacterized protein N657DRAFT_9122	Parathielavia appendiculata	2587402	57	57	19%	7.00E-05	46.48	707	XP_062652007.1
helix-loop-helix DNA-binding domain-containing protein	Xylaria bambusicola	326684	57	57	20%	8.00E-05	42.5	690	XP_047826342.1
uncharacterized protein E0L32_001027	Thyridium curvatum	1093900	56.6	56.6	19%	9.00E-05	45.33	679	XP_030992920.1
Phosphorus acquisition-controlling protein	Colletotrichum gloeosporioides	474922	56.6	56.6	20%	9.00E-05	45.95	683	XP_045263996.1
helix-loop-helix DNA-binding domain-containing protein	Chaetomium tenue	1854479	56.2	56.2	19%	1.00E-04	45.95	554	XP_066648279.1
uncharacterized protein VTJ04DRAFT_3145	Mycothermus thermophilus	85995	56.6	56.6	20%	1.00E-04	40.24	742	XP_069221666.1
uncharacterized protein D7B24_000152	Verticillium nonalfalfae	1051616	56.2	56.2	31%	1.00E-04	38.39	706	XP_028499454.1
phosphorus acquisition-controlling protein	Verticillium dahliae VdLs.17	498257	56.2	56.2	31%	1.00E-04	38.39	706	XP_009655314.1
uncharacterized protein CHGG_00781	Chaetomium globosum CBS 148.51	306901	55.8	55.8	19%	1.00E-04	45.95	560	XP_001220002.1
helix-loop-helix DNA-binding domain-containing protein	Phialemonium atrogriseum	1093897	55.8	55.8	19%	2.00E-04	41.56	684	XP_060285778.1
helix-loop-helix DNA-binding domain-containing protein	Colletotrichum costaricense	1209916	55.5	55.5	20%	2.00E-04	43.42	683	XP_060313964.1
helix-loop-helix DNA-binding domain-containing protein	Colletotrichum tamarilloi	1209934	55.5	55.5	20%	2.00E-04	43.42	683	XP_060384534.1
helix-loop-helix DNA-binding domain-containing protein	Hypoxylon fragiforme	63214	55.5	55.5	19%	2.00E-04	40.79	678	XP_049120896.1
helix-loop-helix DNA-binding domain-containing protein	Truncatella angustata	152316	55.5	55.5	61%	2.00E-04	27.23	687	XP_045964130.1
uncharacterized protein THITE_2113352	Thermothielavioides terrestris NRRL 8126	578455	55.5	55.5	20%	2.00E-04	44.44	700	XP_003652177.1

uncharacterized protein N0V96_001997	Colletotrichum fioriniae	710243	55.5	55.5	20%	2.00E-04	43.42	683	XP_065614771.1
helix-loop-helix DNA-binding domain-containing protein partial	Colletotrichum acutatum	27357	55.1	55.1	20%	3.00E-04	43.42	677	XP_060366756.1
helix-loop-helix DNA-binding domain-containing protein	Colletotrichum godetiae	1209918	55.1	55.1	20%	3.00E-04	43.42	683	XP_060431497.1
helix-loop-helix DNA-binding domain-containing protein	Daldinia caldariorum	326644	55.1	55.1	20%	3.00E-04	42.31	681	XP_047787959.1
Phosphorus acquisition-controlling protein	Daldinia childiae	326645	55.1	55.1	20%	3.00E-04	42.31	681	XP_033433048.1
helix-loop-helix DNA-binding domain-containing protein	Colletotrichum karsti	1095194	55.1	55.1	20%	3.00E-04	44.3	687	XP_038748520.1
Phosphorus acquisition-controlling protein	Madurella fahalii	1157608	55.1	55.1	20%	3.00E-04	41.03	697	XP_070912866.1
helix-loop-helix DNA-binding domain-containing protein	Colletotrichum navitas	681940	54.7	54.7	20%	3.00E-04	43.42	674	XP_060420583.1
uncharacterized protein BZA05DRAFT_125595	Tricharina praecox	43433	54.7	54.7	18%	4.00E-04	52.46	683	XP_051337351.1
Phosphorus acquisition-controlling protein	Scedosporium apiospermum	563466	54.7	54.7	20%	4.00E-04	41.56	693	XP_016644059.1
uncharacterized protein N7483_011706	Penicillium malachiteum	1324776	54.7	54.7	27%	4.00E-04	35.92	681	XP_056941104.1
hypothetical protein QC764_112400	Podospora pseudoanserina	2609844	54.7	54.7	20%	4.00E-04	40.48	726	XP_062805453.1
hypothetical protein QC763_112400	Podospora pseudopauciseta	2093780	54.7	54.7	20%	4.00E-04	40.48	726	XP_062770805.1
helix-loop-helix DNA-binding domain-containing protein	Colletotrichum graminicola M1.001	645133	54.3	54.3	20%	4.00E-04	43.42	674	XP_008093359.1
uncharacterized protein QC762_112400	Podospora pseudocomata	2093779	54.7	54.7	20%	4.00E-04	40.48	726	XP_062748643.1
uncharacterized protein QC761_112400	Podospora bellae-mahoneyi	2093777	54.3	54.3	20%	5.00E-04	40.48	726	XP_062737661.1
helix-loop-helix DNA-binding domain-containing protein	Chaetomium fimeti	1854472	54.3	54.3	19%	5.00E-04	44.74	719	XP_062664661.1
uncharacterized protein PODANS_1_12400	Podospora anserina S mat+	515849	54.3	54.3	20%	5.00E-04	40.48	744	XP_001908909.1
helix-loop-helix DNA-binding domain-containing protein	Lasiosphaeria miniovina	1954250	54.3	54.3	19%	5.00E-04	42.86	698	XP_060290658.1
helix-loop-helix DNA-binding domain-containing protein	Chaetomium strumarium	1170767	54.3	54.3	20%	6.00E-04	42.67	699	XP_062726149.1
Helix-loop-helix DNA-binding domain-containing protein	Colletotrichum higginsianum IMI 349063	759273	53.9	53.9	20%	6.00E-04	43.42	677	XP_018164336.1
Putative myc-type basic helix-loop-helix (bHLH) domain-containin	Colletotrichum destructivum	34406	53.9	53.9	20%	6.00E-04	43.42	678	XP_062775437.1
phosphate-sensing transcription factor PHO4 NDAI_0G02020	Naumovozyma dairenensis CBS 421	1071378	53.9	53.9	35%	7.00E-04	34.26	605	XP_003671220.2
Phosphorus acquisition-controlling protein	Lachnellula hyalina	1316788	53.9	53.9	20%	7.00E-04	38.55	659	XP_031003656.1
HLH transcription factor	Aspergillus saccharolyticus JOP 1030-1	1450539	53.5	53.5	26%	8.00E-04	37.62	701	XP_025428761.1
Phosphorus acquisition-controlling protein	Ceratocystis lukuohia	2019550	53.5	53.5	19%	0.001	43.66	699	XP_070857958.1
uncharacterized protein INS49_008129	Diaporthe citri	83186	53.5	53.5	19%	0.001	42.67	720	XP_043026554.1
helix-loop-helix DNA-binding domain-containing protein	Annulohypoxylon maeteangense	1927788	53.5	53.5	20%	0.001	41.25	684	XP_047824493.1
helix-loop-helix DNA-binding domain-containing protein	Apiospora aurea	335848	53.1	53.1	20%	0.001	41.46	672	XP_066693992.1
helix-loop-helix DNA-binding domain-containing protein	Annulohypoxylon truncatum	327061	53.1	53.1	20%	0.001	41.25	683	XP_047858364.1
helix-loop-helix DNA-binding domain-containing protein	Colletotrichum abscissum	1671311	53.1	53.1	20%	0.001	43.42	683	XP_060390275.1
helix-loop-helix DNA-binding domain-containing protein	Colletotrichum paranaense	1914294	53.1	53.1	20%	0.001	43.42	684	XP_060342349.1
helix-loop-helix DNA-binding domain-containing protein	Colletotrichum scovillei	1209932	53.1	53.1	20%	0.001	43.42	684	XP_035334684.1
uncharacterized protein KY384_008266	Bacidia gigantensis	2732470	53.1	53.1	19%	0.001	43.37	699	XP_044666120.1
helix-loop-helix DNA-binding domain-containing protein	Colletotrichum orchidophilum	1209926	53.1	53.1	20%	0.001	43.42	681	XP_022468412.1
uncharacterized protein PG997_002936	Apiospora hydei	1337664	53.1	53.1	20%	0.001	41.46	612	XP_066670869.1
hypothetical protein AOL_s00083g197	Orbilia oligospora ATCC 24927	756982	52.8	52.8	19%	0.001	47.69	649	XP_011123678.1
uncharacterized protein PG998_002416	Apiospora kogelbergensis	1337665	52.8	52.8	20%	0.001	42.68	707	XP_066737649.1

uncharacterized protein DFL_001941	Arthrobotrys flagrans	97331	52.8	52.8	18%	0.001	50	652	XP_067493272.1
phosphorus acquisition-controlling protein	Diaporthe amygdali	1214568	52.8	52.8	19%	0.001	42.67	704	XP_052998132.1
helix-loop-helix DNA-binding domain-containing protein	Hypoxylon trugodes	326681	52.4	52.4	20%	0.002	41.25	680	XP_051315891.1
uncharacterized protein ONS95_007799	Cadophora gregata	51156	52.4	52.4	20%	0.002	37.8	678	XP_058362637.1
uncharacterized protein N7459_006724 partial	Penicillium hispanicum	1080232	52.4	52.4	27%	0.002	34.95	693	XP_056797891.1
uncharacterized protein PFICI_01435	Pestalotiopsis fici W106-1	1229662	52.4	52.4	20%	0.002	41.98	681	XP_007828207.1
uncharacterized protein JN550_006174	Neoarthrinium moseri	1658444	52.4	52.4	20%	0.002	40.74	681	XP_049171734.1
putative HLH transcription factor (PalcA)	Aspergillus nomiae NRRL 13137	1509407	52	52	26%	0.002	40.96	686	XP_015403083.1
helix-loop-helix DNA-binding domain-containing protein	Durotheca rogersii	419775	52	52	20%	0.003	42.31	681	XP_051375808.1
uncharacterized protein N7477_008027	Penicillium maclennaniae	1343394	50.4	50.4	27%	0.003	33.98	225	XP_056819722.1
uncharacterized protein LTR64_004370	Lithohypha guttulata	1690604	52	52	42%	0.003	33.54	614	XP_064754674.1
uncharacterized protein CTHT_0059650	Thermochaetoides thermophila DSM 1495	759272	52	52	20%	0.003	38.89	823	XP_006696283.1
helix-loop-helix DNA-binding domain-containing protein	Dichotomopilus funicola	1934379	51.6	51.6	20%	0.004	44.44	694	XP_062636635.1
helix-loop-helix DNA-binding domain-containing protein	Colletotrichum lupini	145971	51.6	51.6	20%	0.004	43.42	853	XP_049142153.1
uncharacterized protein BDW74DRAFT_179940	Aspergillus multicolor	41759	51.6	51.6	25%	0.004	39.18	695	XP_073582251.1
HLH helix-loop-helix DNA-binding protein	Venustampulla echinocandica	2656787	51.2	51.2	20%	0.005	36.14	650	XP_031869413.1
PHO4-like protein	Aspergillus mulundensis	1810919	50.8	50.8	25%	0.006	39.18	702	XP_026608378.1
hlh transcription factor	Sporothrix brasiliensis 5110	1398154	50.8	50.8	74%	0.006	27.9	815	XP_040614983.1
hypothetical protein SS1G_12667	Sclerotinia sclerotiorum 1980 UF-70	665079	50.8	50.8	19%	0.006	37.35	634	XP_001586092.1
uncharacterized protein KD926_006598	Aspergillus affinis	1070780	50.4	50.4	26%	0.008	35.64	696	XP_052956681.1
hypothetical protein VTJ83DRAFT_2127	Remersonia thermophila	72144	50.4	50.4	20%	0.008	37.5	746	XP_070868667.1
phosphorus acquisition-controlling protein	Pochonia chlamydosporia 170	1380566	50.4	50.4	19%	0.008	43.48	682	XP_018148550.1
uncharacterized protein LDX57_008057	Aspergillus melleus	138277	50.4	50.4	26%	0.009	35.64	704	XP_045945752.1
uncharacterized protein WHR41_03296	Cladosporium halotolerans	1052096	50.4	50.4	18%	0.009	43.21	738	XP_069230997.1
