# Supplementary material for: Komagataella phaffii Encodes Two Functional Pho4 Transcription Factors
Source: Int J Mol Sci. 2026 Jul 23;27(15):6561. doi: 10.3390/ijms27156561 (PMC13466545; doi:10.3390/ijms27156561)
Supplement: Supplementary file 1 [file ijms-27-06561-s001.zip › Supplementary Table S3A_3B.pdf]

Supplementary Table S3A. Oligonucleotides used in this work

Name	Sequence	Use
**SgRNAs generation**		
**Specific primers**		
1_sgRNA_fw_Pho4(A)-1	TGAAGACGCCATGTGACCCCTGATGAGTCCGTGAGGACGAAACGAG TAAGCTCGTCGGGT	Mutation of *PHO4(A)* at Indel 1
2_sgRNA_fw_Pho4(A)-1	AAACGAGTAAGCTCGTCGGGTCAAAACAACCACCCAGGTTTTAGAG CTAGAAATAGCAAG	Mutation of *PHO4(A)* at Indel 1
1_sgRNA_fw_Pho4(A)-6	TGAAGACGCCATGACCCAGCTGATGAGTCCGTGAGGACGAAACGAG TAAGCTCGTCCTGG	Mutation of *PHO4(A)* at Indel 6
2_sgRNA_fw_Pho4(A)-6	AAACGAGTAAGCTCGTCCTGGGTACATCTCCTCTGGGGTTTTAGAG CTAGAAATAGCAAG	Mutation of *PHO4(A)* at Indel 6
1_sgRNA_fw_Pho4-B-1	TGAAGACGCCATGAATGCACTGATGAGTCCGTGAGGACGAAACGAG TAAGCTCGTCTGCA	Mutation of *PHO4(B)* at Indel 1
2_sgRNA_fw_Pho4-B-1	AAACGAGTAAGCTCGTCTGCATTGGGAGACAACAGTGGTTTTAGAG CTAGAAATAGCAAG	Mutation of *PHO4(B)* at Indel 1
1_sgRNA_fw_Pho4-B-2	TGAAGACGCCATGAATAACCTGATGAGTCCGTGAGGACGAAACGAG TAAGCTCGTCGTTA	Mutation of *PHO4(B)* at Indel 2
2_sgRNA_fw_Pho4-B-2	AAACGAGTAAGCTCGTCGTTATTGTGAAATCTCATGGGTTTTAGAG CTAGAAATAGCAAG	Mutation of *PHO4(B)* at Indel 2
**Common SgRNA primers**		
A_sgRNA_struc_rev	CGCCATGCCGAAGCATGTTGCCCAGCCGGCGCCAGCGAGGAGGCTG GGACCATGCCGGCC	
B_sgRNA_struc_rev	AGAAGACGCAAGCAGTCCAAAGCTGTCCCATTCGCCATGCCGAAGC ATGTTGCCCAGCCG	
C_sgRNA_struc_rev	AGGCTGGGACCATGCCGGCCAAAAGCACCGACTCGGTGCCACTTTT TCAAGTTGATAACG	
D_sgRNA_struc_fw	GTTTTAGAGCTAGAAATAGCAAGTTAAAATAAGGCTAGTCCGTTAT CAACTTGAAAAAGT	

Name	Sequence	Use
**Gene-specific amplification primers**		
Pho4(A) Fw Upst StuI	AGAGGCCTGACCATTACGTTAAGTCGC	Amplification of fragment for *PHO4(A)* including indels 1 & 6
pho4(A)_upARM2-Hygro	CCATGTCGATATCAAGCTTATCTGTTCCATCTTTAGCTATGCTATTTGGAG	Amplification of fragment for *PHO4(A)* including indels 1 & 6
Pho4-B-comp-Fw	GAACCCAACTTCCTTTCCTTCT	Amplification of fragment for *PHO4(B)* including indels 1 & 2
Pho4-B-comp-Rev	GGAATAGCGTCGATCATAGGAG	Amplification of fragment for *PHO4(B)* including indels 1 & 2
**Expression in *S. cerevisiae***		
pho4(A)-expSc-5	CTAGAATTCGACGATAATCCTGACCATTACGTTAAG	Amplification of PHO4(A) ORF for cloning into pWS93
Pho4(A)-expSc-3	TCAGGATCCTACTTTGAACGTTGTTCTTCAAGTTTTTC	Amplification of PHO4(A) ORF for cloning into pWS93
Pho4(B)-expSc-5	ATCGAATTCGATATTGAGGATGAAAATCATGCTAAGAACATC	Amplification of PHO4(B) ORF for cloning into pWS93
Pho4(B)-expSc-3	TACGGATCCTCACTTTTCAAACTCTAATGGACTAATTGAGTC	Amplification of PHO4(B) ORF for cloning into pWS93
3xHA-tetO-5	ACTGGATCCATAATGTACCCATACGATGTTCCTGAC	Amplification of PHO4(A) and PHO4(B) from pWS93-PHO4(A) or (B) for cloning into pCM189 and pCM190
Pho4(A)-tetO-3	ACTCTGCAGCTACTTTGAACGTTGTTCTTCAAG	Amplification of PHO4(A) from pWS93-PHO4(A) for cloning into pCM189 and pCM190
Pho4(B)-tetO-3	ACTGCGGCCGCATCACTTTTCAAACTCTAATGGACTAATTGAGTC	Amplification of PHO4(B) from pWS93-PHO4(B) for cloning into pCM189 and pCM190
pho4_5'_amp	TTTTCTTGCTGCCTTGCCTT	Amplification of *pho4::kanMX4* module from strain ASC15 to generate strain MAC008
pho4_3'_amp	CAACTAGTTCCAACATGCCCA	Amplification of *pho4::kanMX4* module from strain ASC15 to generate strain MAC008

Supplementary Table S3B. HH-sgRNAS-HDV Sequences

Name	Sequence	Target Indel	Construct name
Pho4(A)-CRISP-sgRNA-1	tgaagacgccatgTGACCCCTGATGAGTCCGTGAGGACGAAACGAGTA<br>AGCTCGTCGGGTCAAAACAACCACCCAGGTTTTAGAGCTAGAAATAGC<br>AAGTTAAAATAAGGCTAGTCCGTTATCAACTTGAAAAAGTGGCACCGA<br>GTCGGTGCTTTTGGCCGGCATGGTCCCAGCCTCCTCGCTGGCGCCGGC<br>TGGGCAACATGCTTCGGCATGGCGAATGGGACAGCTTTGGACtgcttg<br>cgtcttct	1	p907_sgRNA-Pho4(A).1
Pho4(A)-CRISP-sgRNA-6	tgaagacgccatgACCCAGCTGATGAGTCCGTGAGGACGAAACGAGTA<br>AGCTCGTCCTGGGTACATCTCCTCTGGGGTTTTAGAGCTAGAAATAGC<br>AAGTTAAAATAAGGCTAGTCCGTTATCAACTTGAAAAAGTGGCACCGA<br>GTCGGTGCTTTTGGCCGGCATGGTCCCAGCCTCCTCGCTGGCGCCGGC<br>TGGGCAACATGCTTCGGCATGGCGAATGGGACAGCTTTGGACtgcttg<br>cgtcttct	6	p907_sgRNA-Pho4(A).6
PHO4-B-sgRNA-1	tgaagacgccatgAATGCACTGATGAGTCCGTGAGGACGAAACGAGTA<br>AGCTCGTCTGCATTGGGAGACAACAGTGGTTTTAGAGCTAGAAATAGC<br>AAGTTAAAATAAGGCTAGTCCGTTATCAACTTGAAAAAGTGGCACCGA<br>GTCGGTGCTTTTGGCCGGCATGGTCCCAGCCTCCTCGCTGGCGCCGGC<br>TGGGCAACATGCTTCGGCATGGCGAATGGGACAGCTTTGGACtgcttg<br>cgtcttct	1	p907_sgRNA-Pho4(B).1
PHO4-B-sgRNA-2	tgaagacgccatgAATAACCTGATGAGTCCGTGAGGACGAAACGAGTA<br>AGCTCGTCGTTATTGTGAAATCTCATGGGTTTTAGAGCTAGAAATAGC<br>AAGTTAAAATAAGGCTAGTCCGTTATCAACTTGAAAAAGTGGCACCGA<br>GTCGGTGCTTTTGGCCGGCATGGTCCCAGCCTCCTCGCTGGCGCCGGC<br>TGGGCAACATGCTTCGGCATGGCGAATGGGACAGCTTTGGACtgcttg<br>cgtcttct	2	p907_sgRNA-Pho4(B).2
